# Deep Learning-Based Stroke Volume Estimation Outperforms Conventional Arterial Contour Method in Patients with Hemodynamic Instability

**DOI:** 10.3390/jcm8091419

**Published:** 2019-09-09

**Authors:** Young-Jin Moon, Hyun S. Moon, Dong-Sub Kim, Jae-Man Kim, Joon-Kyu Lee, Woo-Hyun Shim, Sung-Hoon Kim, Gyu-Sam Hwang, Jae-Soon Choi

**Affiliations:** 1 Biosignal Analysis and Perioperative Outcome Research Laboratory, Department of Anesthesiology and Pain Medicine, Asan Medical Center, University of Ulsan College of Medicine, 88, Olympic-ro 43-gil, Songpa-gu, Seoul 05505, Korea (Y.-J.M.) (J.-M.K.) (G.-S.H.); 2 Health Innovation Bigdata Center, Asan Institute for Lifesciences, 88, Olympic-ro 43-gil, Songpa-gu, Seoul 05505, Korea (H.S.M.) (D.-S.K.) (J.-K.L.); 3 Department of Biomedical Engineering, Asan Medical Center, University of Ulsan College of Medicine, 88, Olympic-ro 43-gil, Songpa-gu, Seoul 05505, Korea

**Keywords:** machine learning, stroke volume, cardiac output, perioperative care, intraoperative monitoring, hemodynamic monitoring

## Abstract

Although the stroke volume (SV) estimation by arterial blood pressure has been widely used in clinical practice, its accuracy is questionable, especially during periods of hemodynamic instability. We aimed to create novel SV estimating model based on deep-learning (DL) method. A convolutional neural network was applied to estimate SV from arterial blood pressure waveform data recorded from liver transplantation (LT) surgeries. The model was trained using a gold standard referential SV measured via pulmonary artery thermodilution method. Merging a gold standard SV and corresponding 10.24 seconds of arterial blood pressure waveform as an input/output data set with 2-senconds of sliding overlap, 484,384 data sets from 34 LT surgeries were used for training and validation of DL model. The performance of DL model was evaluated by correlation and concordance analyses in another 491,353 data sets from 31 LT surgeries. We also evaluated the performance of pre-existing commercialized model (EV1000), and the performance results of DL model and EV1000 were compared. The DL model provided an acceptable performance throughout the surgery (*r* = 0.813, concordance rate = 74.15%). During the reperfusion phase, where the most severe hemodynamic instability occurred, DL model showed superior correlation (0.861; 95% Confidence Interval, (CI), 0.855–0.866 vs. 0.570; 95% CI, 0.556–0.584, *P* < 0.001) and higher concordance rate (90.6% vs. 75.8%) over EV1000. In conclusion, the DL-based model was superior for estimating intraoperative SV and thus might guide physicians to precise intraoperative hemodynamic management. Moreover, the DL model seems to be particularly promising because it outperformed EV1000 in circumstance of rapid hemodynamic changes where physicians need most help.

## 1. Introduction

Assessment of flow-based hemodynamic parameters such as stroke volume (SV) is of great importance in the management of patients in the operating theater. It is believed that SV provides valuable insights into global tissue perfusion and systemic oxygen delivery that can be used to optimize both diagnostic and treatment strategies. Although it is still regarded as the clinical gold standard and offers many kinds of valuable information, the use of pulmonary artery thermodilution is increasingly criticized because of the associated risk of serious complications including pulmonary artery rupture and fatal arrhythmia [1]. Because the thermodilution method requires highly invasive instrumentation, its application is not clinically feasible.

Recently, less invasive commercially available arterial pressure waveform-based SV measuring methods (e.g., EV1000; Edwards Lifescience, Irvine, CA, USA) have become popular [2]. The arterial waveform analysis generally is considered to be minimally invasive and provides continuous data that enable a prompt response to be initiated. However, there have been several concerns regarding such devices, including the need for manual calibration, and unreliable estimation during hemodynamic instability [2,3]. Traditional pressure waveform analysis has been based purely on the morphology of the pressure contour. Furthermore, errors arise from the calibration method, which relies on the use of fixed arterial properties. Those shortcomings are evident when it is used in patients with extreme levels of arterial resistance such as those seen in septic shock, end-stage liver disease, or chronic renal failure [4,5,6]. Consequently, the deficiencies in methods of monitoring SV were always found during periods of hemodynamic instability, which is exactly the point where SV monitoring becomes essential [7]. Inaccurate surrogate measures of SV can lead to misdiagnosis, incorrect clinical treatment, and misinterpretation of patient response. 

Deep learning (DL) is now evolving as a rising technology in variety of medical fields [8,9]. Convolutional neural network (CNN) is the main reason for the recent overwhelming success of neural network in solving various image classification problems [10,11]. The key difference of CNN is that it has convolution/pooling layers and fully connected layers, while conventional neural network models only have fully connected layers [8]. In the medical field, medical image classification and diagnosis problems have been solved with great accuracy by CNN model [12], but the application of the CNN model to time-series data is yet limited.

In this study, we propose a novel method of tracking SV through a CNN model using arterial blood pressure (ABP) waveform data during the LT surgery as input data and SV measured via pulmonary artery thermodilution method as a gold standard referential. We also compared the performance of DL model and EV1000, which is pre-existing ABP-based commercialized SV predicting model.

## 2. Methods

### 2.1. Data Preparation 

The study was approved by the Institutional Review Board of the Asan Medical Center (No. 2018-1163), and written informed consent from participants was waived. The prospectively recorded hemodynamic and medical data of 74 consecutive liver transplantation (LT) surgeries performed from February 2018 to April 2018 in our institution were collected and analyzed in a retrospective fashion. The anesthetic management, perioperative patient care, and vital sign data recruitment for LT surgery were performed according to the standard institutional protocol of the Asan Medical Center, which has been previously described in detail [13,14,15,16]. In brief, anesthesia was induced with thiopental sodium, vecuronium, and fentanyl. After endotracheal intubation, anesthesia was maintained with sevoflurane or desflurane in a 50% oxygen/air mixture and continuous infusion of vecuronium and fentanyl. The hemodynamic parameters including electrocardiogram (ECG), pulse oximetry, core temperature, capnometer, and radial and femoral arterial pressure were continuously monitored. Pulmonary arterial pressure (PAP) and inferior vena cava (IVC) pressure were monitored via Swan-Ganz and femoral vein catheterization, respectively. SV was continuously monitored using Vigillence II (SV measuring device using pulmonary artery thermodilution method, Edwards Lifescience, Irvine, CA, USA) and EV1000 (ABP waveform-based SV measuring methods).

Data were collected from the electronic medical record and LT database. The full scale hemodynamic variables of all recipients were routinely recorded during LT with a computerized data acquisition software called Vital Recorder [17]. The variables recorded included continuous ECG, ABP, PAP, central venous pressure (CVP), pulse oximetry, airway pressure and capnography, all of which were digitalized at a different sampling rate.

### 2.2. Machine Learning Based SV Estimation

**Figure 1:**
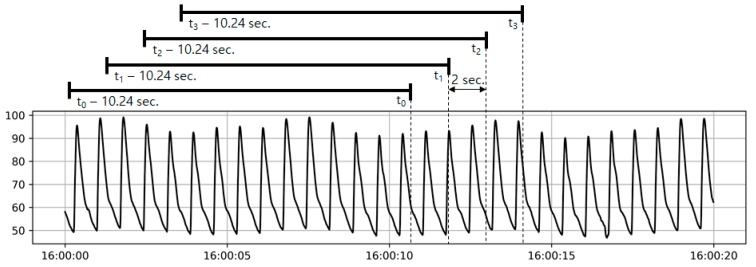
Representative figure describes how an input record is generated. For a time point t_0_, an input vector consists of arterial blood pressure (ABP) waveform between t_0_–10.24 sec, and t_0_. Since the waveform data is collected at a sampling rate of 100 Hz, each input vector has 1024 ABP values. SV_PAC_ measured at t_0_ is regarded as a reference and SV_EV1000_ measured at t_0_ is regarded as a competitor. Data records were generated once 2 seconds so neighboring records overlap each other.

In this paper, an input vector was composed of ABP waveform data and a response variable was composed of SV value. ABP input vector of 1024 measured BP values from arterial line method sampled at a rate of 100 Hz. A data record is composed of the input vector and corresponding SV values of the same time point (Figure 1). We used the SV value measured by pulmonary artery catheterization (PAC) as the reference. Then, we applied the model to a separate testing data set that was not used in the training stage to predict SV value. Finally, we compared the predicted SV value with the PAC SV value to evaluate the performance of the proposed method. 

### 2.3. Convolutional Neural Network (CNN)

A CNN is a machine learning model that has recently shown an overwhelming performance especially in various image classification problems including medical image classification [10]. In an SV prediction problem, it is expected that we can utilize the advantageous characteristics of CNN since additional features that are overlooked in physiological models may exist in the shape of an ABP waveform. Although previous research was mostly focused on a classification problem [18], current study is a regression problem. Furthermore, image data is two-dimensional, whereas ABP waveform is 1D data. Therefore, we build a CNN model as follows. First, an input vector was composed of 1024 samples gained at 100 Hz of sampling rate (over 10.24 seconds) since an input vector should include at least one respiratory cycle to consider the variation associated with respiration. Then, merging a gold standard SV and corresponding 10.24 seconds of ABP waveform as an input/output data set with 2-senconds of slide overlapping. A block composed of a convolutional layer and a max pooling layer so that the length of an input vector was decreased by a factor of 2 in each block. There are eight blocks in total and the last layer is a fully connected layer to get a single value. An input of a block was composed of an output of the last block and an input of the last block. Although the idea was originally proposed by He et al. [19], we modified it slightly for our problem. The cost function was defined as a RMS error between the predicted SV value and the true SV value. The model was optimized using an Adam optimizer and was implemented using Tensorflow library 1.4 and Python 3.5.

### 2.4. Interpersonal Scale Variation in Training Data

Even if the shape and scale of ABP waveform is the same, SV value may vary with height, weight, BMI, and Child-Turcotte-Pugh class of the patient. In this case, response variables (SV value) from different patients are different from each other even for the same ABP waveform. It becomes more difficult to train a model tasking this factor into consideration. In this research, we proposed a variable called individual scale coefficient (ISC) to overcome the problem.

Our use of ISC was as follows. Let SV_CNN_ be a SV value predicted by the proposed CNN model. Then a scaled SV value that reflects interpersonal variation is defined as SV_scaled_ = SV_CNN_ * w_i_ + b_i_, where i is a patient and w_i_ and b_i_ are individual scale coefficients for that patient i. These coefficients are shared within a patient and vary between patients. When we train a model, we give w_i_ = 1 and b_i_ = 0 for all i initially and optimize these variables with the CNN part simultaneously to find optimum ISCs.

### 2.5. Dataset, Model Training, and Post-Processing of Predicted SV Values

Of the 74 consecutive patients enrolled, nine patients were excluded from analysis due to lack of intraoperative recording of hemodynamic data. Then, the remaining 65 patients were analyzed for developing a SV prediction model. Patients were divided into training set group (*n* = 34) and testing set group (*n* = 31). The data from patients of the training set group were used to build a SV prediction model, while the data from the patients of the testing set group were used to compare the model performance with the existing models (Vigilance II and EV1000). Each data record was composed of radial ABP waveform of 10.24 seconds and its corresponding SV values from Vigilance II and EV1000. The time required for building a deep learning model was on, an average, less than an hour with a Linux server with 4 GTX-1080Ti GPUs. In the experiment, we applied the model to radial ABP data obtained from LT surgery and compared the predicted result with SV values measured by PAC. To evaluate performance of the proposed model, we also compared the predicted result with SV values estimated by EV1000, which is a commercialized SV estimator based on ABP waveform data.

### 2.6. Statistical Analysis

Variables are expressed as numbers (percentages), mean ± standard deviation, or median (interquartile range) as appropriate. Analyses between groups were performed using student’s *t*-test, Mann–Whitney U test, analysis of variance, logistic regression, or Kruskal–Wallis test for continuous variables and χ^2^ test or Fisher’s exact test for categorical variables, as appropriate. Linear regression analysis was used to evaluate the relationship between the reference values (SV_PAC_) and the testing values (SV_EV1000_ and SV_DL_). Bland-Altman plot was used to calculate the bias and limits of agreement of SV_EV1000_ and SV_DL_ [20]. The trend analysis was performed using a quadrant plot. In the paper, an error margin of 10% was used to calculate the concordance rate. Performance error (PE, (measured SV – predicted SV)/predicted SV) was derived to evaluate the model performance of EV1000 and DL model [21]. Median performance error (MDPE), median absolute performance error (MDAPE), and root mean square error (RMSE) were also calculated. All statistical variables were compared between the EV1000 and DL models. In addition, model comparisons were performed separately for each phase of the liver transplantation surgery (pre-anhepatic, anhepatic, reperfusion, and post-reperfusion phases). Statistical analysis was performed using MedCalc 18.6 (MedCalc Software, Ostend, Belgium) and Python 3.5 (Python Software Foundation). *P* value < 0.05 was considered to be statistically significant.

## 3. Results

The baseline characteristics of the patients are displayed in Table 1. The patients’ demographic characteristics did not statistically differ among patients of training, validation, and testing sets. The total 975,737 matched data records during a time window of 542.1 hours were analyzed in our study (484,384 matched data records from 34 patients of the training/validation set group and 491,353 matched data records from 31 patients of the testing set group). Each phase of liver transplantation (pre-anhepatic, anhepatic, reperfusion, and post-reperfusion) presents different hemodynamic characteristics. All hemodynamic variables including SV_PAC_ changed significantly as the surgery proceeded (Table 2).

The SV data from all measurement techniques (PAC, EV1000, and DL model) are summarized in Table 2. Using PAC, the gold standard method, a broad range of SV values were measured during the operation (84.8 ± 24.7 mL, range 30–222 mL). The EV1000 and DL models resulted in a comparable SV throughout the surgery (91.5 ± 33.1 mL, range 17–214 mL and 84.8 ± 24.7 mL, range 24.1–183.9 mL, respectively).

**Figure 2:**
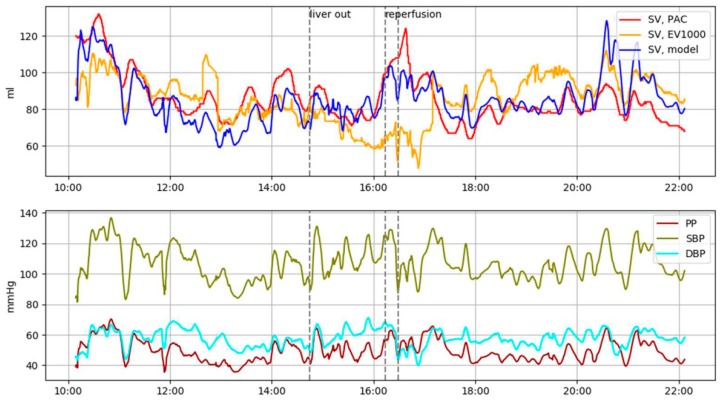
Representative plot of intraoperative tracking of stroke volume (SV) trends of Vigilance, EV1000 and proposed method during liver transplantation surgery. Note that proposed method (blue) shows a similar trend to Vigilance (red), while EV1000 (orange) fails to follow SV trend especially during the before and after the reperfusion phase (15:30–17:00).

Table 3 demonstrates the performances of each SV measurement technique (EV1000 and DL model) compared to the SV_PAC_. Notably, the prediction performance of the DL model was superior to that of the EV1000 technique during the reperfusion phase, which is the phase with the most drastic and severe hemodynamic instability (Figure 2). During the reperfusion phase, the correlation coefficient was significantly higher in the DL model than in the EV1000 one (0.861; 95% CI, 0.855–0.866 vs. 0.570; 95% CI, 0.556–0.584, *P* < 0.001). The DL model also showed a higher concordance rate than the EV1000 model (90.62% vs. 75.76%). The proposed model also outperformed the older model during other phases: correlation coefficients were higher than EV1000 (0.837; 95% CI, 0.836–0.838 vs. 0.821; 95% CI, 0.820–0.823 in pre-anhepatic phase and 0.828; 95% CI, 0.827–0.829 vs. 0.795; 95% CI, 0.793–0.797 in post-reperfusion phase. All *P* < 0.001).

**Figure 3:**
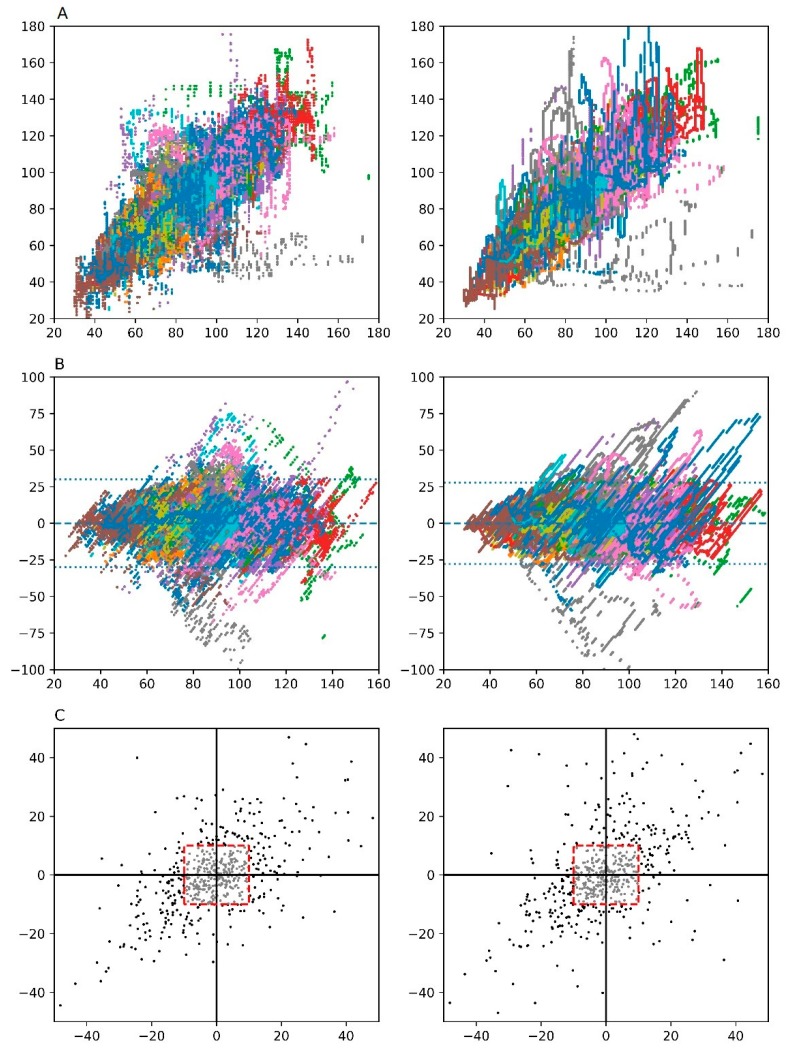
Scatter (**A**), Bland Altman (**B**) and Four-quadrant (**C**) plot analysis between the target stroke volume (SV) and predicted SV. Predicted SVs are EV1000 (left) and proposed model (right), respectively. Each color represents each cases. A central exclusion zone (red square) is shown in Four-quadrant plot.

The overall performance of the DL model was significantly better than that of the EV1000 one. The correlation coefficients were 0.840 (95% CI, 0.839–0.841) and 0.813 (95% CI, 0.812–0.814) in total when comparing SV_PAC_ with SV_DL_ and SV_EV1000_, respectively (both *P* < 0.001, Table 3, Figure 3). Bland-Altman analysis revealed lower SD of the difference during all the phases when comparing SV_PAC_ with SV_EV1000_ and SV_DL_, respectively. Trends analysis for SV_PAC_ with both SV_EV1000_ and SV_DL_ demonstrated comparable concordance rate (74.15% vs. 77.74%).

We also evaluated the predicted result based on PE and RMSE, and the result was concordant with the correlation analysis (Table S1). During all four phases, the proposed method showed a lower absolute PE. (0.1131 vs. 0.1233 in pre-anhepatic phase, 0.1292 vs. 0.1401 in anhepatic phase, 0.1134 vs. 0.2067 in reperfusion phase, and 0.1188 vs. 0.1345 in post-reperfusion phase. All differences were significant.) The overall absolute PE was also lower (0.1176 vs. 0.1317).

## 4. Discussion

In the present study, we proposed a stroke volume estimation method using arterial blood pressure waveform through a deep-learning approach. The DL model showed better concordance in predicting SV compared to the conventional method during certain phases of surgery. Notably, the DL model outperformed EV1000 during reperfusion phase when the most serious hemodynamic changes occur. One point that should be emphasized in our proposed DL model is that the feature extraction from the arterial waveform was not achieved by the clinician but by the DL model itself. Given the superior performance of the DL model over the EV1000, it is plausible that the DL model might locate the hidden features of the arterial waveform, which were not unveiled by the clinicians.

The proposed DL model showed better performance in clinically critical situations. During the reperfusion phase when the most severe hemodynamic instability occurs, EV1000 addressed poor performance compared to the performances shown in the other phases of surgery. In contrast, the DL model achieved a consistent performance throughout the entire procedure. The major purpose of the advanced cardiovascular monitoring, including SV estimation, is to maintain optimal perfusion pressure and flow during the most unstable hemodynamic situations [22]. However, preexisting commercialized SV-estimating models have common shortcomings that call their validity into question.

Previous studies consistently raised a concern of limited accuracy in situations of major cardiovascular changes and extremes of individual vascular tone [5,6,23]. The post-reperfusion phase of LT surgery is the most challenging period because of the severe hemodynamic instability resulting from extremely low systemic vascular resistance or sometimes even from concomitant low cardiac contractility [14]. Those pre-existing devices did not show their best performance at the time when they were needed most. We are inclined to place emphasis on the fact that such major fluctuations in vascular tone might negatively influence the performance of the EV1000 method as evidenced by several studies [2,5]. In contrast, within a systemic vascular resistance range of 400 to 1300 dyne∙s/cm^5^, the correlation coefficient was 0.840, which implies that a consistent performance was provided by the DL model. Considering the fact that the current DL model was developed based on the hemodynamic data of specific patients undergoing LT, it is plausible that our model fits well into the situation of patients with low systemic vascular resistance.

The use of the deep-learning method to build certain kinds of prediction model is increasing in the medical field. Patients’ actual SV was predicted with a multi-layer neural network, which showed less prediction error than the conventional model and arterial contour analysis. Conventional methods to calculate or estimate SV are mostly based on features extracted from ABP waveform such as systolic BP, diastolic BP, pulse pressure, and systolic area. However, there could be information loss since these features are abstract. Another problem of these approaches is that it is difficult to continually improve the performance of the method. For example, in a certain case, where the method worked poorly, a more generalized model should be built, which also caters for these atypical cases. The main advantage of our deep learning model architecture is that its application is suitable for various scenarios. The high dimensionality problems in traditional covariate modeling can be eliminated because our model directly relates covariates with effect. Furthermore, it does not require any manual feature extraction, and its global effect could be easily expanded by just adding more cases to the training set. For example, it is known that the current SV estimators work incorrectly in the particular case of patients with sepsis. In a machine learning-based approach, the problem can easily be solved by just adding data from patients with sepsis to the training dataset. In this paper, the proposed method outperformed an existing method, especially in the reperfusion phase. This benefit stems from the fact that the input includes data from such unusual phases so that the model can predict SV value for such kinds of waveform more accurately.

The proposed DL model has a few novel features from an engineering perspective as well as a clinical one. There have been previous studies that report the application of a CNN model to a signal classification problem [24,25,26,27]. However, few papers took a CNN approach to solve a regression problem. In the present paper, it was shown that a CNN model can also be applied to a regression problem. Another challenge in the solution of the SV estimation enigma is interpersonal variation: each patient has his/her own SV scale, which is unique to that particular individual. In this paper, we used an ISC for calibration. A similar approach has been used previously [28], but we have also shown that these factors can be taken into account simultaneously during model training and applied to similar problems.

The current study has several limitations. First, there are limitations regarding the deep learning approach. Generalization of the deep learning model is dependent on the training data set. Our dataset consisted of patients undergoing LT, which is a major operative procedure, so that most of the patients suffered from various cardiovascular diseases and altered vascular characteristics. In patients having untrained rare conditions, the performance might be impaired. Expanding the patient subset to a wider group of populations and including various pathological conditions such as sudden cardiovascular collapse, together with repeated machine learning from the accumulated data, would lead to a more robust model. Another inherent limitation of DL model is that interpreting the inside mechanism/algorithm of deep learning is not feasible. In the deep-learning model, a relation between input and output is represented as a form of complicated and non-linear weighted network structure. More specifically, the weight value of each node is neither informative nor plausible. All problems related to overfitting or retrospective design may be partly solved by increasing the case numbers in further prospective studies. Second, heart-lung interaction created by mechanical ventilator causes hemodynamic swings in 5–6 seconds periods. Thus, it might have little influence on the precision of the DL model. However, to minimize the influence of heart-lung interaction factor, 10.24 sec length of arterial waveform was defined as a data set that at least covers one ventilator cycle. Third, the DL model might not cover broad spectrum of patients underwent LT surgery. Because of the small number of patient inclusion, our data set cannot represent the whole spectrum of LT patients. However, regarding the SV, a single patient shows a broad range of SV during the surgery regardless of MELD score or the cause for the surgery. Our current data set seems to include enough SV data so that it showed better prediction performance over pre-existing commercialized model (EV1000), although only 34 patients were included. Fourth, the reperfusion phase is where DL model shows superior outcome over EV1000, but it is only a small portion of the surgery and the amount of data was less than 1% of total. Nonetheless, we believe the information provided by DL model during reperfusion is very critical because most of intraoperative morbidity and mortality may occur during this period. Future studies showing whether the DL model actually improve hemodynamic management or clinical outcomes is warranted to justify the use of this new technology.

In conclusion, we showed the possible usefulness of the new SV estimation model which was created by a DL method. Through a modified CNN approach, time series data of ABP waveform were successfully evolved into a clinically relevant hemodynamic management tool with outstanding performance. Applying the DL method in monitoring cardiovascular hemodynamics seems promising since it could improve the accuracy of measures to improve hemodynamic function in a wide range of patients, a problem that the pre-existing methods have not solved.

## Figures and Tables

**Table 1:** Patient characteristics for training and testing set.

	Training Set (*n* = 33)	Testing Set (*n* = 31)	Total Set (*n* = 64)	*P*-Value
**Demographics**				
Age (years)	54.7 ± 9.5	55.3 ± 8.1	55.0 ± 8.8	0.800
Sex (male)	66.7%	61.3%	64.1%	0.851
Weight (kg)	64.7 ± 13.7	70.2 ± 13.3	67.4 ± 13.7	0.111
Body mass index (kg/m^2^)	23.5 ± 4.0	25.7 ± 5.2	24.5 ± 4.7	0.056
MELD score	12 (8–15)	17 (10–21)	14 (9–21)	0.333
CTP score	7 (6–8)	9 (6–10.5)	7 (6–9.3)	0.239
grade A	42.4%	29.0%	35.9%	0.392
grade B	36.4%	38.7%	37.5%	1.000
grade C	21.2%	32.3%	26.6%	0.474
**Causes for liver transplantation**				
Hepatitis B virus related liver cirrhosis	54.5%	38.7%	46.9%	0.309
Hepatitis C virus related liver cirrhosis	0%	16.1%	7.8%	0.053
Alcoholic liver cirrhosis	24.2%	25.8%	25.0%	1.000
Hepatocellular carcinoma	54.5%	45.2%	50.0%	0.617
Others	15.2%	12.9%	14.1%	1.000
**Operation type**				
Living donor	87.9%	90.3%	89.1%	1.000
Deceased donor	12.1%	9.7%	10.9%	1.000
**Underlying disease**				
Diabetes mellitus	24.2%	22.6%	23.4%	1.000
Hypertension	24.2%	22.6%	23.4%	1.000
**Medication**				
Beta blocker	21.2%	25.8%	23.4%	0.890
Diuretics	42.4%	48.4%	45.3%	0.820

Values are expressed as mean ± standard deviation, median (interquartile range), or percent. MELD, model for end-stage liver disease; CTP, Child-Turcotte-Pugh.

**Table 2:** Trends of hemodynamic parameters during the liver transplantation.

	Phases of Liver Transplantation					*P* for Trend
	Pre-Anhepatic	Anhepatic	Reperfusion	Post-Reperfusion	Overall	
**Duration (min, %)**	7042 (43.0 %)	2080 (12.7%)	295 (1.8%)	6962 (42.5%)	16378 (100%)	
**Blood pressure (mmHg)**						
Systolic	110.1 ± 16.3	104.4 ± 16.3	97.2 ± 17.6	105.0 ± 14.2	107.0 ± 15.7	<0.001
Diastolic	56.0 ± 8.6	55.0 ± 7.9	48.1 ± 7.1	53.2 ± 7.8	54.5 ± 8.3	<0.001
**Heart rate (bpm)**	82.8 ± 16.0	88.2 ± 18.5	86.5 ± 18.4	83.4 ± 17.5	83.8 ± 17.1	<0.001
**Stroke volume (mL/beat)**						
SV_PAC_	88.5 ± 23.5	75.3 ± 24.0	85.7 ± 25.2	83.8 ± 25.1	84.8 ± 24.7	<0.001
SV_EV1000_	91.5 ± 30.3	85.9 ± 33.5	91.3 ± 36.5	93.2 ± 35.4	91.5 ± 33.1	<0.001
SV_DL_	87.9 ± 23.4	76.3 ± 22.1	83.7 ± 26.4	84.2 ± 25.9	84.8 ± 24.7	<0.001
**Stroke volume index (mL/beat/m^2^)**						
SVI_PAC_	50.8 ± 14.8	42.6 ± 14.3	49.0 ± 14.0	47.6 ± 13.4	48.4 ± 14.4	<0.001
SVI_EV1000_	51.8 ± 16.1	48.2 ± 18.4	51.7 ± 19.2	52.9 ± 19.8	51.8 ± 18.2	<0.001
SVI_DL_	50.3 ± 14.5	43.1 ± 13.4	47.7 ± 14.2	47.9 ± 14.2	48.4 ± 14.4	<0.001
**Systemic vascular resistance (dyne∙s/cm^5^)**	850.0 ± 331.3	910.3 ± 393.0	749.9 ± 272.2	856.9 ± 290.7	858.8 ± 323.6	<0.001
**Stroke volume variation by SV_EV1000_ (%)**	7.6 ± 4.1	10.2 ± 7.0	8.4 ± 6.0	9.8 ± 5.7	8.9 ± 5.4	<0.001

Values are expressed as mean ± standard deviation or numbers (percent). SV_PAC_, SVI_PAC_, SV_EV1000_, and SVI_EV1000_ refer to stoke volume (index) measured by pre-existing monitoring devices that using pulmonary artery catheter (a gold standard method, Vigilance II, Edward Lifesciences) and radial arterial catheter (EV1000, Edward Lifesciences), respectively. SV_DL_ and SVI_DL_ refer to a stoke volume (index) value predicted by a deep-learning algorithm using radial arterial waveform of the patients. SV, stoke volume; SVI, stroke volume index; PAC, pulmonary arterial catheter; DL, deep-learning.

**Table 3:** Comparative analyses of stroke volume measurements.

Phases	Data Records (*n*)	Linear Regression Analysis			Bland-Altman Analysis				Four-Quadrant Analysis	
		Pearson Correlation, *r* (95% CI)			Bias (mL)		95% Limits of Agreement (mL)		Concordance Rate (%)	
Comparison with SV_PAC_ as Standard Reference										
		**SV_EV1000_**	**SV_DL_**	***P* Value**	**SV_EV1000_**	**SV_DL_**	**SV_EV1000_**	**SV_DL_**	**SV_EV1000_**	**SV_DL_**
**Overall**	491,353	0.813 (0.812–0.814)	0.840 (0.839–0.841)	<0.001	Na	Na	−29.52 ~ +29.52	−27.36 ~ +27.36	74.15%	77.74%
**Pre-anhepatic**	211,265	0.821 (0.820–0.823)	0.837 (0.836–0.838)	<0.001	0.96	−0.63	−26.75 ~ +28.67	−26.87 ~ +25.61	75.00%	75.61%
**Anhepatic**	62,391	0.866 (0.864–0.868)	0.865 (0.863–0.867)	0.48	2.73	0.99	−21.50 ~ +26.96	−22.77 ~ +24.75	82.14%	95.65%
**Reperfusion**	8,841	0.570 (0.556–0.584)	0.861 (0.855–0.866)	<0.001	−4.66	−2.01	−49.13 ~ +39.81	−28.76 ~ +24.74	75.76%	90.62%
**Post-reperfusion**	208,856	0.795 (0.793–0.797)	0.828 (0.827–0.829)	<0.001	−1.59	0.43	−33.03 ~ +29.85	−28.91 ~ +29.77	70.43%	74.80%
		**SVI_EV1000_**	**SVI_DL_**	***P* Value**	**SVI_EV1000_**	**SVI_DL_**	**SVI_EV1000_**	**SVI_DL_**	**SVI_EV1000_**	**SVI_DL_**
**Overall**	491,353	0.827 (0.826-0.828)	0.848 (0.848–0.849)	<0.001	Na	Na	−16.58 ~ +16.58	−15.52 ~ +15.52	74.58%	77.42%
**Pre-anhepatic**	211,265	0.847 (0.846–0.848)	0.860 (0.859–0.861)	<0.001	0.38	−0.45	−15.46 ~ +16.22	−15.64 ~ +14.74	75.00 %	75.61%
**Anhepatic**	62,391	0.882 (0.880–0.884)	0.878 (0.876–0.880)	0.002	1.49	0.55	−11.94 ~ +14.92	−12.88 ~ +13.98	82.14%	95.65%
**Reperfusion**	8,841	0.561 (0.546–0.575)	0.861 (0.856–0.867)	<0.001	−2.65	−1.29	−27.35 ~ +22.05	−15.70 ~ +13.12	75.76%	91.62%
**Post-reperfusion**	208,856	0.789 (0.788–0.790)	0.817 (0.816–0.819)	<0.001	−0.73	0.32	−18.25 ~ +16.79	−16.10 ~ +16.74	71.55%	74.59%

SV_PAC_, SVI_PAC_, SV_EV1000_, and SVI_EV1000_ refer to stoke volume (index) measured by pre-existing monitoring devices that using pulmonary artery catheter (a gold standard method, Vigilance II, Edward Lifesciences) and radial arterial catheter (EV1000, Edward Lifesciences), respectively. SV_DL_ and SVI_DL_ refer to a stoke volume (index) value predicted by a deep-learning algorithm using radial arterial waveform of the patients. SV, stoke volume; SVI, stroke volume index; PAC, pulmonary arterial catheter; DL, deep-learning; Na, not applicable.

## Supplementary Materials

The following are available online at https://www.mdpi.com/2077-0383/8/9/1419/s1, Table S1: Predicted result based on performance error and root mean square error.
