# Supplementary material for: Deep Learning-Based Stroke Volume Estimation Outperforms Conventional Arterial Contour Method in Patients with Hemodynamic Instability
Source: J Clin Med. 2019 Sep 9;8(9):1419. doi: 10.3390/jcm8091419 (PMC6780281; doi:10.3390/jcm8091419)
Supplement: Supplementary file 1 [file jcm-08-01419-s001.pdf]

Supplementary Table

Predicted result based on performance error and root mean square error

	Data Records	MDPE		MDAPE		APE			RMSE	
		EV1000	DL model	EV1000	DL model	EV1000	DL model	P value	EV1000	DL model
**Overall**	491,353	0.0032	0.0000	0.0966	0.0861	0.1317 ± 0.1257	0.1176 ± 0.1143	<0.001	15.06	13.90
**Pre-anhepatic**	211,265	0.0032	−0.0160	0.0863	0.0802	0.1233 ± 0.1251	0.1131 ± 0.1162	<0.001	14.17	13.39
**Anhepatic**	62,391	0.0399	0.0161	0.0990	0.0898	0.1401 ± 0.1342	0.1292 ± 0.1199	<0.001	12.65	11.84
**Reperfusion**	8,841	−0.0575	−0.0526	0.1497	0.0911	0.2067 ± 0.2097	0.1134 ± 0.0990	<0.001	23.17	12.90
**Post-reperfusion**	208,856	−0.0065	0.0173	0.1054	0.0903	0.1345 ± 0.1173	0.1188 ± 0.1110	<0.001	16.12	14.97

EV1000 refer to pre-existing stroke volume monitoring device that using arterial blood pressure from arterial catheter. DL, deep-learning; PE, (measured SV-predicted SV) / predicted SV; MDPE (median performance error), median of PE; MDAPE, median of absolute PE; RMSE, root mean square error.
